# Therapeutic potential of C1632 by inhibition of *SARS-CoV-2* replication and viral-induced inflammation through upregulating *let-7*

**DOI:** 10.1038/s41392-021-00497-4

**Published:** 2021-02-22

**Authors:** Chen Xie, Yanlian Chen, Dongling Luo, Zhen Zhuang, Heping Jin, Haoxian Zhou, Xiaocui Li, Haijun Lin, Xiaohui Zheng, Jing Zhang, Peihui Wang, Jincun Zhao, Yong Zhao, Hui Huang

**Affiliations:** 1 Key Laboratory of Gene Engineering of the Ministry of Education, School of Life Sciences, Sun Yat-sen University, Guangzhou, Guangdong China; 2 Cardiovascular Department, The Eighth Affiliated Hospital, Sun Yat-sen University, Shenzhen, Guangdong China; 3 State Key Laboratory of Respiratory Disease, Guangzhou Institute of Respiratory Disease, The First Affiliated Hospital of Guangzhou Medical University, Guangzhou, Guangdong China; 4 Xiamen Innodx Biotech Co., Ltd., Xiamen, Fujian China; 5 School of Pharmaceutical Sciences, Wenzhou Medical University, Wenzhou, Zhejiang China; 6 Advanced Medical Research Institute, Cheeloo College of Medicine, Shandong University, Jinan, Shandong China

**Keywords:** Molecular medicine, Infectious diseases

**Dear Editor,**

The COVID-19 pandemic caused by *SARS-CoV-2* has led to acute respiratory distress syndrome (ARDS) with a high rate of death. An excessive inflammatory response, caused by virus infection, is associated with severe clinical manifestations that may lead to death of patients.^1^ Therefore, the blockage of virus replication and suppression of hyper-inflammatory response are beneficial for COVID-19 treatment. However, the drug targeting both virus and hyper-inflammation, as far as we know, is not available yet.

MicroRNAs (miRNAs) are small, non-coding RNAs that play regulatory roles in gene expression by targeting their mRNA. Several miRNAs have been identified to negatively affect HIV-1 or HCV by directly targeting the viral RNA genome and/or by repressing the expression of virus-dependent cellular cofactors.^2^ *Let-7* is miRNA containing 13 family members in human cells. It has been previously reported that *let-7* is capable to attenuate the virulence of influenza virus that causes pneumonia. We speculated that *let-7* may have a similar function on COVID-19 by targeting SARS-CoV-2. To test this idea, bioinformatics analysis was first performed to identify putative target sites on *SARS-CoV-2* genome. Two *let-7* binding sites with sequences complementary to seed region of *let-7-3p* were identified that are located at coding sequences of S and M protein of *SARS-CoV-2*, respectively (Supplementary Fig. S1a, b). Experimentally, we demonstrated that *let-7d*, *let-7e*, *let-7f*, *let-7g*, *let-7i*, and *miR-98* were able to significantly suppress the expression of S protein (Fig. 1a), whereas *let-7b*, *let-7c*, *let-7g*, *let-7i*, and *miR-98* inhibited M protein expression (Fig. 1b).

**Fig. 1:**
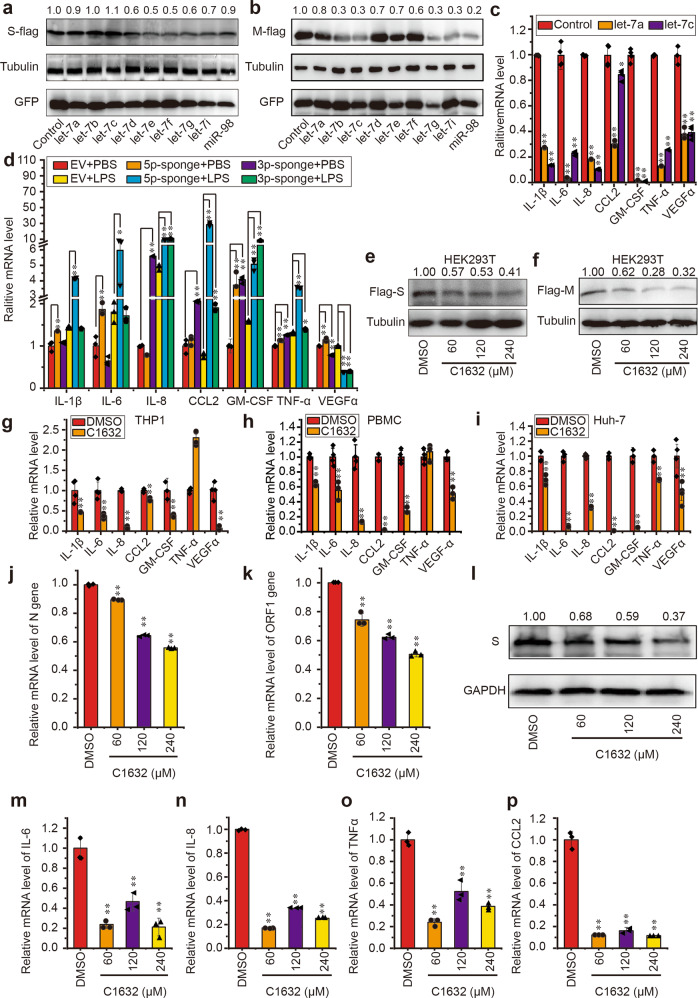
Validation of C1632 as a potential anti-SARS-CoV-2 drug that suppresses both virus replication and viral-induced inflammation by upregulating let-7. *Let-7* inhibits exogenous expression of S protein (**a**) and M protein (**b**) in HEK293T cells. GFP was cloned into vector to ensure the equal transfection/expression efficiency. Scramble sequence was used as a control. **c** The expression level of IL-1β, IL-6, IL-8, CCL2, GM-CSF, TNF-α, and VEGFα were downregulated by overexpressed *let-7a* and *let-7c* in THP1 cells. **d** *let-7-5p* and *let-7-3p* sponges increased the expression level of multiple inflammatory factors in THP1 cells. *let-7* stimulator C1632 inhibited exogenous expression of S (**e**) and M (**f**) protein in HEK293T cells in a concentration dependent manner. **g**–**i** The expression of IL-1β, IL-6, IL-8, CCL2, GM-CSF, and VEGFα were downregulated by C1632 in THP1 derived macrophages (**g**) and PBMCs (**h**). **i** The expression of IL-1β, IL-6, IL-8, CCL2, GM-CSF, TNF-α, and VEGFα were downregulated by C1632 in Huh-7 cells. The RNA level of N (**j**) and ORF1 (**k**) were suppressed by C1632 in a dosage dependent manner in *SARS-CoV-2* infected Huh-7 cells. **l** C1632 treatment decreased S protein level in *SARS-CoV-2* infected Huh-7 cells (western blot). The expression level of IL-6 (**m**), IL-8 (**n**), TNF-α (**o**) and CCL2 (**p**) were downregulated by C1632 in *SARS-CoV-2* infected Huh-7 cells

It has been reported that *let-7a* and *let-7c* inhibit the expression of IL-6, a typical inflammatory factor induced by *SARS-Cov-2*,^3^ raising the possibility that upregulation of *let-7* may downregulate inflammatory factors, except for IL-6, helping to attenuate the cytokine storm caused by *SARS-Cov-2*. To test this hypothesis, *pri-let-7a* and *pri-let-7c* were overexpressed in THP1 cells, respectively (Supplementary Fig. S2a&b). Interestingly, *let-7a* or *let-7c* not only reduced mRNA level of IL-6, but also significantly decreased the expression of many other *SARS-Cov-2* associated cytokines and chemokines including IL-1β, IL-8, CCL2, GM-CSF, TNF-α, and VEGFα (Fig. 1c). Using *let-7* 5p sponge and 3p sponge that significantly reduced the level of matured *let-7-5p* and *let-7-3p* (Supplementary Fig. S2c), we observed that *let-7-5p* sponge significantly increased the expression of IL-1β, IL-6, IL-8, GM-CSF, and TNF-α, whereas *let-7-3p* sponge increased the expression of IL-8, CCL2, GM-CSF, and TNF-α in both untreated and LPS-stimulated THP1 cells (Fig. 1d). These results implied that *let-7* is capable for broad-spectrum inhibition of cellular inflammatory reaction.

A small molecule C1632 (*N*-Methyl-*N*-[3-(3-methyl[1,2,4]triazolo[4,3*-*b] pyridazin-6-*yl*)phenyl]acetamide) has been identified to block the interaction between LIN28 and *pri/pre*-*let-7*, thus promoting the maturation of *let-7*.^3^ Here, we demonstrated that treatment with C1632 (60, 120 and 240 μM) for 24 h is capable to greatly reduce the expression of S and M protein, which is associated with a significant increase of *let-7-5p* and *let-7-3p* in HEK293T cells (Fig. 1e, f and Supplementary Fig. S3a). This anti-inflammation effect of C1632 was also tested in human lung epithelial cancer cell line A549, liver cancer cell line Huh-7, leukemic cell line THP1, and peripheral blood mononuclear cell (PBMC). Our results demonstrated that C1632 significantly increases the level of *let-7-5p* and *let-7-3p* in these cells (Supplementary Fig. S3b–e). Accordingly, the expression level of many inflammatory cytokines and chemokines including IL-1β, IL-6, IL-8, CCL2, GM-CSF, and VEGFα decreased in all tested cell lines (Fig. 1g–i and Supplementary Fig. S4a).

To imitate the situation in vivo, we examined anti-inflammation effect of C1632 in LPS-stimulated PBMCs. We observed that C1632 significantly decreases the expression level of many inflammatory cytokines and chemokines stimulated by LPS, including IL-1β, IL-6, IL-8, CCL2, GM-CSF, TNF-α, and VEGFα (Supplementary Fig. S4b and Supplementary Fig. S3f).

To extend our understanding of how many inflammatory factors are affected by C1632, THP1 cells were treated with LPS in the presence or absence of C1632, and secreted cytokines were determined by Luminex assay. The result showed that C1632 treatment leads to more than 2.5 folds decrease of secreted factors including IL-1β, IL-1α, IL-1 RA, IP-10, IL-6, IL-10, IL-18, GM-CSF, and CCL2 (Supplementary Table. S1). It is worth noting that secreted IL-8 are slightly increased upon C1632 treatment, which is inconsistent with observed decrease in their mRNA level (Fig. 1g), underlying mechanism remained to be elucidated.

Given that M and S protein are essential structural components for *SARS-CoV-2* assembly, budding and infection, it is conceivable to speculated that increased level *let-7* by C1632 would reduce M and S protein, thus suppressing virus replication. Indeed, when *SARS-CoV-*2 infected human Huh-7 cells (MOI = 0.1) were treated with C1632 for 48 h, virus load, which is indicated by expression level of virus’s N and ORF1 genes, was significantly decreased (Fig. 1j, k). This is consistent with observed decrease of S protein (Fig. 1l). Moreover, we observed that while *SARS-Cov-2* infection stimulates the expression of many inflammatory factors in Huh-7 cells (Supplementary Fig. S5), C1632 treatment leads to significant decrease of IL-6, IL-8, TNF-α and chemokine CCL2 (Fig. 1m–p). These results demonstrated dual functions of C1632 as an inhibitor of *SARS-CoV-2* replication and anti-inflammation reagent.

It has been reported that NF-κB upregulates the expression of LIN28, leading to a low level of *let-7*. Meanwhile, *let-7* could suppress the expression of IL-6 that activates NF-κB by stimulating STAT3.^3^ Thus, NF-κB/LIN28/*let-7*/IL-6/STAT3 forms a positive feedback loop during cellular inflammation. It is likely that increased level of *let-7* by C1632 may break this feedback loop, reducing inflammation levels. Consistently, both overexpression of *let-7* and C1632 are capable of suppressing the expression of multiple inflammatory factors involving inflammatory factor storms induced by *SARS-CoV-2*. Moreover, C1632 is a putative inhibitor of bromodomain proteins, which promote the transcription of inflammation-related genes via binding acetylated histones.^3^ Therefore, C1632 may suppress inflammation responses by inhibiting the activity of bromodomain proteins.

So far, there is no specific drug for treatment of *SARS-CoV-2*. Here, we reported that *let-7*, a miRNA that is ubiquitously expressed in human cells, blocks *SARS-CoV-2* replication by targeting S and M protein. Meanwhile, *let-7* suppresses the expression of multiple inflammatory factors including IL-1β, IL-6, IL-8, CCL2, GM-CSF, TNF-α, and VEGFα. More importantly, C1632, a small molecule serving as a *let-7* stimulator, is capable to upregulate the expression of *let-7*, thus reducing viral replication and secretion of inflammatory cytokines. It has been previously demostrated that C1632 displays a low toxicity for cultured cells and mice and has been potented to treat pet’s noise and thunderstorm phobias.^4,5^ The safety and beneficial effect of C1632 on inhibiting *SARS-CoV-2* replication and suppressing viral-induced inflammation should be highly emphasized. Further research on the safety and effectiveness of C1632 will help promote its clinical application.

## Supplementary information

Supplementary Information

## Data Availability

Plasmids encoding *let-7*-5p sponge (P20227) and *let-7*-3p (P20228) sponge are available from MiaoLing Plasmid Sharing Platform.
