# Supplementary material for: Therapeutic potential of C1632 by inhibition of SARS-CoV-2 replication and viral-induced inflammation through upregulating let-7
Source: Signal Transduct Target Ther. 2021 Feb 22;6:84. doi: 10.1038/s41392-021-00497-4 (PMC7897876; doi:10.1038/s41392-021-00497-4)
Supplement: Supplementary file 1 — Supplementary Information [file 41392_2021_497_MOESM1_ESM.docx]

Supplementary Materials for

Therapeutic Potential of C1632 by Inhibition of SARS-CoV-2 Replication and Viral-induced Inflammation through upregulating Let-7

Chen Xie^1,7^, Yanlian Chen^1,7^, Dongling Luo ^2^, Zhen Zhuang^3^, Heping Jin^1^, Haoxian Zhou^1^, Xiaocui Li^1^, Haijun Lin^4^, Xiaohui, Zheng^5^, Jing Zhang^6^, Peihui Wang^6^, Jincun Zhao^3^, Yong Zhao^1^*, Hui Huang^2^*

^1^Key Laboratory of Gene Engineering of the Ministry of Education, School of Life Sciences, Sun Yat-sen University, 510006, Guangzhou, Guangdong, China;

^2^Cardiovascular Department, The Eighth Affiliated Hospital, Sun Yat-sen University, 518000, Shenzhen, Guangdong, China;

^3^State Key Laboratory of Respiratory Disease, Guangzhou Institute of Respiratory Disease, The First Affiliated Hospital of Guangzhou Medical University, 510182, Guangzhou, Guangdong, China;

^4^Xiamen Innodx Biotech Co., Ltd., No.124, Xinyuan Road, Haicang District, 361022, Xiamen, Fujian, China;

^5^School of Pharmaceutical Sciences, Wenzhou Medical University, 325035, Wenzhou, Zhejiang, China;

^6^Advanced Medical Research Institute, Cheeloo College of Medicine, Shandong University, 250012, Jinan, Shandong, China;

^#^These authors contributed equally to this work.

* To whom correspondence should be addressed:

HH, E-mail:[huangh8@mail.sysu.edu.cn](mailto:huangh8@mail.sysu.edu.cn)

Or YZ, E-mail: [zhaoy82@mail.sysu.edu.cn](mailto:zhaoy82@mail.sysu.edu.cn)

**This Word file includes:**

Supplementary Fig. S1 to S5

Supplementary Table S1 to S2

MATERIALS AND METHODS

REFERENCES


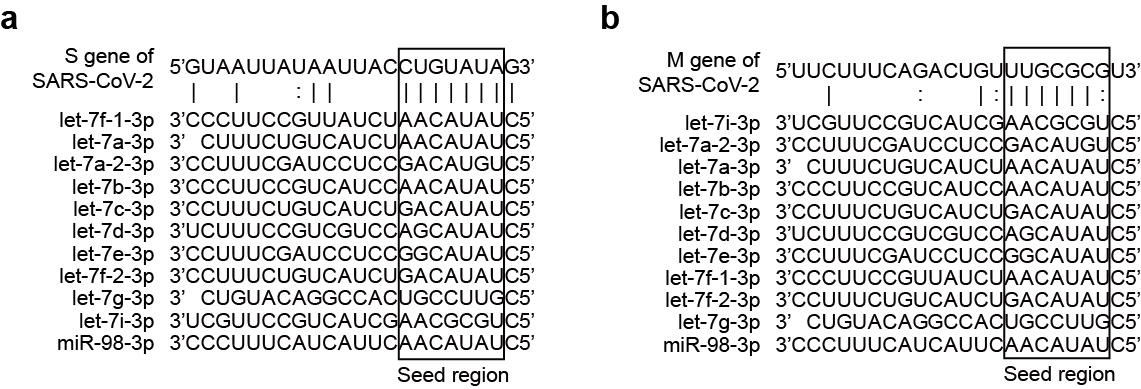


**Supplementary Fig. S1** Predicted target sequences of *let-7* in S (A) and M (B) gene of *SARS-CoV-2*.


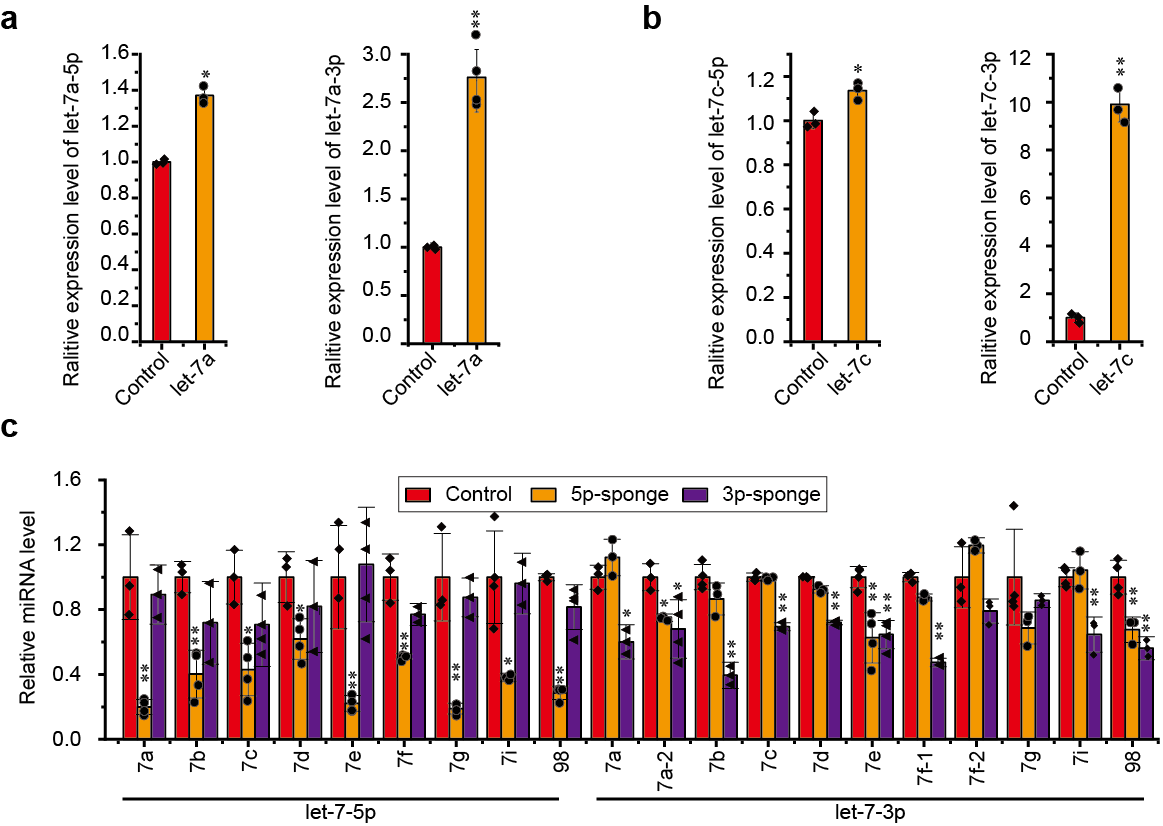


**Supplementary Fig. S2** qRT-PCR detection of *let-7* levels. (A, B) *let-7a* and *let-7c* were over-expressed in THP1 cells. (C) *let-7-5p* and *let-7-3p* sponges suppressed the expression level of *let-7-5p* or *let-7-3p* in THP1 cells.


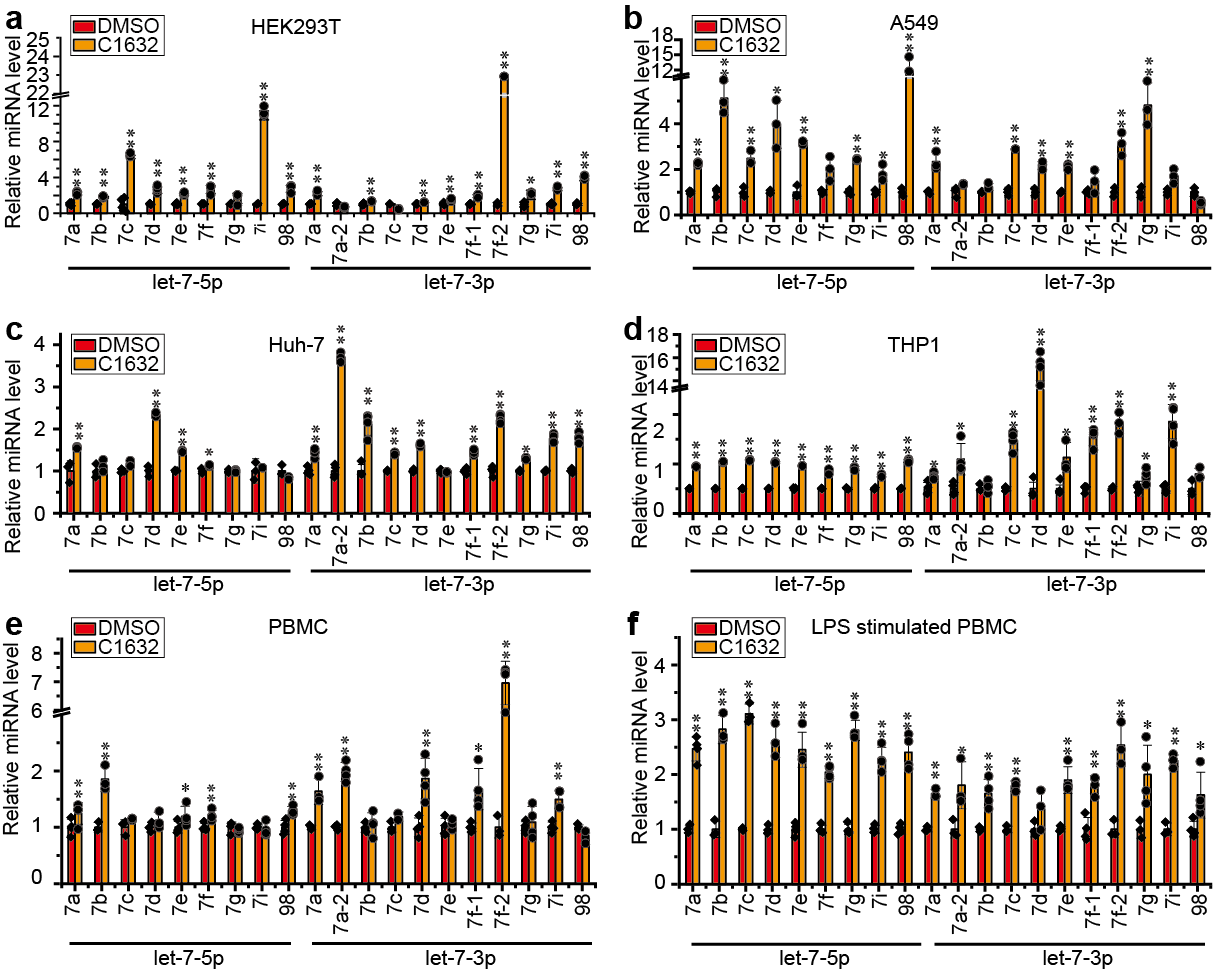


**Supplementary Fig. S3** C1632 stimulates the expression of *let-7*. C1632 up-regulated the expression of *let-7-5p* and *let-7-3p* in HEK293T cells (A), A549 cells (B), Huh-7 cells (C), THP1 derived macrophages (D), PBMCs (E) and LPS stimulated PBMCs (F).


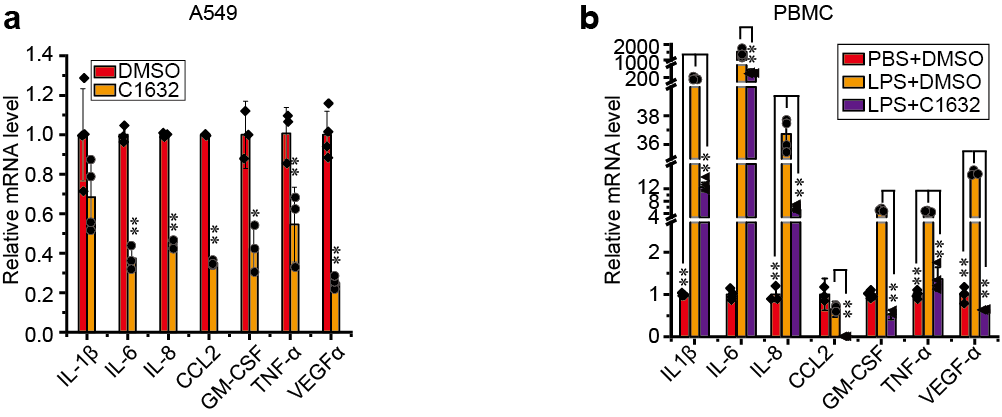


**Supplementary Fig. S4** The expression level of IL-1β, IL-6, IL-8, CCL2, GM-CSF, TNF-α and VEGFα were down-regulated by C1632 in A549 cells (A) and LPS stimulated PBMCs (B)*.*

**
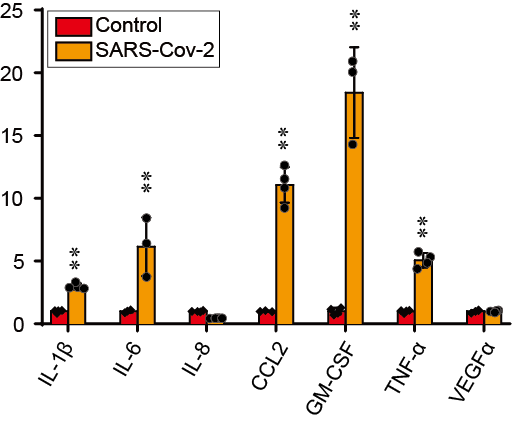
**

**Supplementary Fig. S5** *SARS-Cov-2* induce the expression of inflammatory factors in Huh7 cells.

**Supplementary Table S1.** Comparison of secreted inflammatory factors in THP1-derived macrophages treated with or without C1632.

| **Inflammatory factors** | **DMSO (pg/ml)** | **C1632 (pg/ml)** | **Ratio** |
| --- | --- | --- | --- |
| IL-10 | 312.6 | 2.8 | 0.01 |
| IP-10 | 4487 | 42.3 | 0.01 |
| IL-1 RA | 69245 | 2108 | 0.03 |
| CCL2 | 4257 | 275.1 | 0.06 |
| IL-6 | 273.1 | 19 | 0.07 |
| IL-18 | 163.1 | 21.5 | 0.13 |
| IL-1 beta | 6531 | 1492 | 0.23 |
| GM-CSF | 269.3 | 65.4 | 0.24 |
| IL-1 alpha | 14.6 | 5 | 0.34 |
| IL-22 | 28.8 | 13.3 | 0.46 |
| RANTES | 466.3 | 420.6 | 0.90 |
| SDF-1alpha | 8076 | 7609 | 0.94 |
| GRO-alpha | 2416 | 2823 | 1.17 |
| MIP-1 alpha | 841.5 | 994.2 | 1.18 |
| Eotaxin | 12.8 | 17.3 | 1.35 |
| TNF-alpha | 12566 | 17121 | 1.36 |
| IL-5 | 10 | 14.5 | 1.45 |
| IL-4 | 10.9 | 17.4 | 1.60 |
| IL-8 | 3441 | 7213 | 2.10 |
| IL-7 | 1 | 2.1 | 2.10 |
| IL-2 | 13.4 | 30.6 | 2.28 |
| IL-13 | 4.9 | 14.4 | 2.94 |
| IL-27 | <8 | 83.1 | ND |
| IL-12p70 | <3.3 | <3.3 | ND |
| IL-17A | <1.1 | 9 | ND |
| IL-31 | <3.9 | <3.9 | ND |
| IFN-gamma | <8.8 | <8.8 | ND |
| MIP-1 beta | >45918 | >45918 | ND |
| IFN-alpha | <0.1 | 0.8 | ND |
| IL-9 | <1.4 | 4.9 | ND |
| TNF-beta | <4.7 | <4.7 | ND |
| IL-23 | <4.9 | <4.9 | ND |
| IL-15 | <1.9 | 13.3 | ND |
| IL-21 | <5 | 5.2 | ND |

**Table S2.** The q-RT-PCR primers used in this research.

| Primer name | Sequence |
| --- | --- |
| U6-RT | CTCAACTGGTGTCGTGGAGTCGGCAATTCAGTTGAGAAAAATATG |
| U6-F | CTCGCTTCGGCAGCACA |
| U6-R | AACGCTTCACGAATTTGCGT |
| hsa-let-7a-2/7c/7e-3p RT | CTCAACTGGTGTCGTGGAGTCGGCAATTCAGTTGAGGGAAAGCT |
| hsa-let-7a-3p RT | CTCAACTGGTGTCGTGGAGTCGGCAATTCAGTTGAGGAAAGACA |
| hsa-let-7b/7f-1-3p RT | CTCAACTGGTGTCGTGGAGTCGGCAATTCAGTTGAGGGGAAGGC |
| hsa-let-7d-3p RT | CTCAACTGGTGTCGTGGAGTCGGCAATTCAGTTGAGAGAAAGGC |
| hsa-let-7f-2-3p RT | CTCAACTGGTGTCGTGGAGTCGGCAATTCAGTTGAGGGAAAGAC |
| hsa-let-7g-3p RT | CTCAACTGGTGTCGTGGAGTCGGCAATTCAGTTGAGGCAAGGCA |
| hsa-let-7i-3p RT | CTCAACTGGTGTCGTGGAGTCGGCAATTCAGTTGAGAGCAAGGC |
| hsa-miR-98-3p RT | CTCAACTGGTGTCGTGGAGTCGGCAATTCAGTTGAGGGGAAAGT |
| hsa-let-7a-2-3p F | CCAGCTGGGCTGTACAGCCTCCTAG |
| hsa-let-7a-3p F | CCAGCTGGGCTATACAATCTACTGT |
| hsa-let-7b-3p F | CCAGCTGGGCTATACAACCTACTGC |
| hsa-let-7c-3p F | CCAGCTGGGCTGTACAACCTTCTAG |
| hsa-let-7d-3p F | CCAGCTGGGCTATACGACCTGCTGC |
| hsa-let-7e-3p F | CCAGCTGGGCTATACGGCCTCCTAG |
| hsa-let-7f-1-3p F | CCAGCTGGGCTATACAATCTATTGC |
| hsa-let-7f-2-3p F | CCAGCTGGGCTATACAGTCTACTGT |
| hsa-let-7g-3p F | CCAGCTGGGCTGTACAGGCCACTGC |
| hsa-let-7i-3p F | CCAGCTGGGCTGCGCAAGCTACTGC |
| hsa-miR-98-3p F | CCAGCTGGGCTATACAACTTACTAC |
| Stem-Loop Uni-Reverser | CTGGTGTCGTGGAGTCGGCAATT |
| GAPDH-F | CCCATGTTCGTCATGGGTGT |
| GAPDH-R | TGGTCATGAGTCCTTCCACGATA |
| N-F | GGGGAACTTCTCCTGCTAGAAT |
| N-R | CAGACATTTTGCTCTCAAGCTG |
| ORF1ab-F | CCCTGTGGGTTTTACACTTAA |
| ORF1ab-R | ACGATTGTGCATCAGCTGA |
| IL-6-F | CCTGAACCTTCCAAAGATGGC |
| IL-6-R | TTCACCAGGCAAGTCTCCTCA |
| IL-8-F | ACTGAGAGTGATTGAGAGTGGAC |
| IL-8-R | AACCCTCTGCACCCAGTTTTC |
| TNF-α-F | GAGGCCAAGCCCTGGTATG |
| TNF-α-R | CGGGCCGATTGATCTCAGC |
| IL-1β-F | TTCGACACATGGGATAACGAGG |
| IL-1β-R | TTTTTGCTGTGAGTCCCGGAG |
| CCL2-F | CAGCCAGATGCAATCAATGCC |
| CCL2-R | TGGAATCCTGAACCCACTTCT |
| VEGFα-F | CCTCCACCATGCCAAGTGGT |
| VEGFα-R | ATCGCATCAGGGGCACACAG |

## MATERIALS AND METHODS

### Plasmid construction

pCDNA3.1-2019-nCoV-S-3×FLAG (Cat#P13282) was from MiaoLing Plasmid Sharing Platform. pcDNA6B-nCoV-M-FLAG was a gift from Dr. Wang Peihui at Shandong University. *Let-7* expression vectors were gifts from Dr.Jiang Songshan at Sun Yat-sen University.^1^

*Let-7-3p* sponge and *let-7-5p* sponge were designed according to the method previously reported.^2^ Sponges sequence were synthesized at GENEWIZ and subcloned into pLVX-AcGFP-N1 (Clontech, Palo Alto, CA, USA). The constructed plasmids were preserved in MiaoLing Plasmid Sharing Platform.

### Cells and virus culture

HEK293T, A549, Vero E6 cells and Huh-7 cells were cultured in DMEM (high glucose) medium (Invitrogen, Carlsbad, CA, USA). THP1 and PBMC (Leide Biosciences Co., Ltd, Guangzhou, China) were cultured in RPMI-1640 medium (Invitrogen, Carlsbad, CA, USA). Mediums were supplemented with 10% (V/V) fetal bovine serum (FBS, Invitrogen, Carlsbad, CA, USA) and antibiotics (100U/mL of penicillin and 100µg/ml of streptomycin, Invitrogen, Carlsbad, CA, USA).

Clinically isolated *SARS-CoV-2* viruses (Genebank accession no. MT123290.1) were propagated in Vero E6 cells. Viral titer was determined by 50% tissue culture infective dose (TCID50) according to the cytopathic effect measured by Reed-Muench method.^3^ Cells were either mock-infected or infected with *SARS-CoV-2*, as previously described.^4^ Virus was allowed to adsorb at room temperature for 1 hour before incubation at 37 °C for indicated time. All infection experiments were performed in a biosafety level-3 (BLS-3) laboratory.

### Drug treatment

C1632 was synthesized as previously reported.^5^ C1632 and PMA (P1680, MDBio Inc, Qingdao, China) were dissolved in DMSO. TNF-α (P01375, Novoprotein Scientific Inc, Shanghai, China) was dissolved in distilled water. LPS (L2880, Sigma, St. Louis, MO, USA) was dissolved in PBS. All these drugs were stored at -20℃.

A549, Huh-7, HEK293T and HEK293T expressing exogenous S and M protein were seeded into 6-well plates at a density of 2×10^5^ per well. Next day, cells were treated with 240μM C1632 for 48h (DMSO serves as a control) and assayed for q-RT-PCR or western blot analysis.

THP1 cells were seeded into 12-well plates at density of 5×10^5^ per well. 100ng/ml PMA was added to medium to induce differentiation of THP1 into macrophages. After 24h, fresh medium was replaced and cells were treated with 240μM C1632 for 48h (DMSO serves as a control). To stimulate the inflammatory response, 100ng/ml LPS or 50ng/ml TNF-α was added to medium for 12h before q-RT-PCR and ELISA analysis.

PBMCs were seeded into 12-well plates at density of 5×10^6^ per well and treated with 240μM of C1632 for 24h. 100ng/ml LPS was added to medium for 6-8h to stimulate PBMCs before q-RT-PCR assay.

### Transfection

HEK293T cells were seeded in a 12-well plate at density of 2×10^5^ per well. After 24h, 500ng vector expressing S or M protein genes and 500ng vector expressing *let-7s* were mixed, diluted with 100μl opti-MEM medium (Invitrogen, Carlsbad, CA, USA), mixed with 3μl 1mg/ml PEI solution (#23966, Polyscience Inc., Warrington, PA, USA), vortexed, and incubated at room temperature for 15 min. Mixture was then added to prepared HEK293T cells.

### RNA Extraction and Real-time Quantitative RT-PCR

Total RNA was extracted from cells using the MagZol reagent (Magen, Guangzhou, China) according to manufacturer’s instructions. miRNAs were reverse-transcribed to DNA by Rever-Tra-Ace-α- Transcriptase (Toyobo, Tokyo, Japan). Stem-loop method was employed to validate the expression level of *let-7s*.^6^ U6 snRNA served as an internal reference gene. mRNA was reverse-transcribed by TransScript® One-Step gDNA Removal and cDNA Synthesis SuperMix (AT311, Transgen Biotech, Beijing, China). cDNA was amplified by PCR using the 2×SYBR qPCR mix (KT Life technology Co., Ltd, Shenzhen, China). Q-PCR was performed with a LightCycler 480 Real-Time PCR system (Roche, Rotkreuz, Switzerland). Data was analyzed with the comparative Ct (2-ΔΔCt) method. All experiments were performed in triplicate.

*Let-7-5p* specific primer sequences were designed as described previously^6^. *Let-7-3p* specific primers and qPCR primers for mRNA detection were listed in supplemental **Table S2**.

### Immuno Blot

HEK293T cells were lysed with SDS lysis buffer. *SARS-CoV-2*-infected Huh-7 cells were lysed with MagZol reagent, RNA and protein were then purified respectively. Purified protein was dissolved in SDS lysis buffer. Protein samples were boiled and loaded onto a 10% SDS–polyacrylamide gel electrophoresis (PAGE) for separation. Proteins were transferred to PVDF membrane (Pall, MA, USA), incubated with primary and second antibody, and visualized by enhanced chemiluminescence detection reagents (Pierce, Rockford, IL, USA). Rabbit anti-*SARS-Cov-2* S (Cat#40592-T62) and Rabbit anti-*SARS-Cov-2*-NP (Cat#40143-R019) were purchased from Sino Biological. Mouse anti-β-actin was purchased from Santa Cruz Biotechnology Inc. Rabbit anti-GFP (D110008) were purchased Sangon Biotech. Goat-anti-mouse and goat-anti-rabbit was purchased from MultiSciences. Mouse anti-flag (Cat#MY1906) was purchased from MerryBio. Rabbit anti-GFP (D110008) was purchased from Sangon Biotech (Shanghai) Co., Ltd.

### Luminex

Luminex method was used for high-throughput determination of protein levels of inflammatory factors and cytokines. In detail, 3×10^6^ THP1 were seeded at 6cm cell culture dish. PMA with a final concentration of 100 ng/ml was added and incubated overnight to induce differentiation of THP1 into macrophages. Next day, replace with fresh medium and add 240 μM C1632 to continue culturing for 2 days (DMSO is used as a control). 100 ng/ml LPS were used to stimulate the inflammation. 12h after stimulation, the culture medium was collected and subjected to Luminex assay by Kingmed Diagnostics Co., Ltd.

**LIN28 Inhibitor C1632 Synthesis**

*N*-methyl-*N*-[3-(3-methyl[1,2,4] triazolo [4,3-b]-pyridazin-6-*yl*) phenyl ] acetamide

(CL285032 also named C1632) was synthesized according to a literature reported procedure (Scheme 1) . Commercially available 1, 2 and 4 were purchased from J&K Chemical Company. The synthesis steps are as follows: (1) 1 and 2 were reacted overnight in boiling methylbenzene. (2) 3 was obtained from flash chromatography using 6 % (v/v) MeOH/CH_2_Cl_2_. Purified 3 was reacted with 4 in TAF at 45℃ overnight. (3) 5 was obtained from flash chromatography using 6 % (v/v) MeOH/CH_2_Cl_2_. Purified 5 was reacted overnight in boiling acetic acid. The CL285032 was obtained by flash chromatography using MeOH/CH2Cl2 (0–15%) as a light-yellow solid: ^1^H NMR (CDCl_3_, d, ppm): 8.42 (d, J = 9.6 Hz, 1H), 8.11 (br s, 2H), 7.98 (d, J = 9.6 Hz, 1H), 7.66 (t, J = 7.8 Hz, 1H), 7.57 (d, J = 6.8 Hz, 1H), 3.24 (s, 3H), 2.78 (s, 3H), 1.85 (s, 3H); LC–MS (ESI): 282.0; Elemental analysis (calcd, found for C_15_H_15_N_5_O): C (64.04, 63.79), H (5.37, 5.42), N (24.90, 25.01), O (5.69, 5.78).

Scheme 1. The synthesis of CL285032. ^7^

## REFERENCES

1. Xie, C. et al. MDM4 regulation by the let-7 miRNA family in the DNA damage response of glioma cells*.* *FEBS Letters* **589**, 1958-1965 (2015).

2. Ebert, M.S. et al., MicroRNA sponges: competitive inhibitors of small RNAs in mammalian cells*.* *Nat Methods* **4**, 721-726 (2007).

3. Wang, M. et al. Remdesivir and chloroquine effectively inhibit the recently emerged novel coronavirus (2019-nCoV) in vitro*.* *Cell Research* **30**, 269-271 (2020).

4. Runfeng, L. et al. Lianhuaqingwen exerts anti-viral and anti-inflammatory activity against novel coronavirus (SARS-CoV-2)*.* *Pharmacol Res* **156**, 104761 (2020).

5. Chen, Y. et al. LIN28/let-7/PD-L1 Pathway as a Target for Cancer Immunotherapy*.* *Cancer Immunology Research* **7**, 487-497 (2019).

6. Wang, Y. et al. Quantification of distinct let-7 microRNA family members by a modified stem-loop RT-qPCR*.* *Molecular medicine reports* **17**, 3690-3696 (2018).

7. Ghidu VP. et al. A new and efficient synthetic route for the anxiolytic agent CL285032. *Bioorganic & Medicinal Chemistry Letters* **21**, 259-61 (2011).
